# Two Green Micellar HPLC and Mathematically Assisted UV Spectroscopic Methods for the Simultaneous Determination of Molnupiravir and Favipiravir as a Novel Combined COVID-19 Antiviral Regimen

**DOI:** 10.3390/molecules27072330

**Published:** 2022-04-04

**Authors:** Yasmine Ahmed Sharaf, Sami El Deeb, Adel Ehab Ibrahim, Ahmed Al-Harrasi, Rania Adel Sayed

**Affiliations:** 1 Analytical Chemistry Department, Faculty of Pharmacy, Zagazig University, Zagazig 44511, Egypt; yasminesharaf2009eg@gmail.com (Y.A.S.); raniaadelsayed@gmail.com (R.A.S.); 2 Institute of Medicinal and Pharmaceutical Chemistry, Technische Universitaet Braunschweig, 38106 Braunschweig, Germany; 3 Natural and Medical Sciences Research Center, University of Nizwa, P.O. Box 33, Birkat Al Mauz, Nizwa 616, Oman; adel.ehab@pharm.psu.edu.eg (A.E.I.); aharrasi@unizwa.edu.om (A.A.-H.); 4 Analytical Chemistry Department, Faculty of Pharmacy, Port-Said University, Port Said 42526, Egypt

**Keywords:** molnupiravir, favipiravir, COVID-19 regimen, micellar liquid chromatography, UV–VIS Spectrophotometry

## Abstract

Following the spread of the COVID-19 pandemic crisis, a race was initiated to find a successful regimen for postinfections. Among those trials, a recent study declared the efficacy of an antiviral combination of favipiravir (FAV) and molnupiravir (MLP). The combined regimen helped in a successful 60% eradication of the SARS-CoV-2 virus from the lungs of studied hamster models. Moreover, it prevented viral transmission to cohosted sentinels. Because both medications are orally bioavailable, the coformulation of FAV and MLP can be predicted. The developed study is aimed at developing new green and simple methods for the simultaneous determination of FAV and MLP and then at their application in the study of their dissolution behavior if coformulated together. A green micellar HPLC method was validated using an RP-C18 core-shell column (5 μm, 150 × 4.6 mm) and an isocratic mixed micellar mobile phase composed of 0.1 M SDS, 0.01 M Brij-35, and 0.02 M monobasic potassium phosphate mixture and adjusted to pH 3.1 at 1.0 mL min^−1^ flow rate. The analytes were detected at 230 nm. The run time was less than five minutes under the optimized chromatographic conditions. Four other multivariate chemometric model methods were developed and validated, namely, classical least square (CLS), principal component regression (PCR), partial least squares (PLS-1), and genetic algorithm–partial least squares (GA–PLS-1). The developed models succeeded in resolving the great similarity and overlapping in the FAV and MLP UV spectra unlike the traditional univariate methods. All methods were organic solvent-free, did not require extraction or derivatization steps, and were applied for the construction of the simultaneous dissolution profile for FAV tablets and MLP capsules. The methods revealed that the amount of the simultaneously released cited drugs increases up until reaching a plateau after 15 and 20 min for FAV and MLP, respectively. The greenness was assessed on GAPI and found to be in harmony with green analytical chemistry concepts.

## 1. Introduction

Following the wide spread of the SARS-CoV-2 virus in the beginning of 2020, a pandemic crisis emerged where the world nations exerted tremendous efforts to contain the spread of COVID-19. COVID-19 causes severe respiratory syndrome leading to a serious disease that affects multiple organs in the human body, including the kidneys and liver, as well as nervous system [1]. Since the start of the pandemic, drug makers have been racing to develop new vaccinations and antiviral molecules against SARS-CoV-2, the causative virion; however, this was not an easy process. Despite the presence of several globally approved vaccines recently, the inactivity against new variants of SARS-CoV-2, and the issues of low protection of immune-compromised patients together with the high magnitude of population resistance caused by people’s distrust made the vaccination process challenging [2,3]. Moreover, some scientists were worried about the possibility of integration of the viral mRNA-based genes into the human genome [4]. A recent in vitro study [5] examined such previous worries, and it unfortunately augmented the people’s distrust about the marketed vaccines. The mRNA vaccine, BNT162b2, which was developed by Pfizer was reported to be uptaken into human liver cells and incorporated within the host DNA [5]. On the other hand, the development and approval process of new drug molecule is strict and requires a long time to establish its safety profile before human consumption [6]. Therefore, scientists focused their trials on repurposing previously approved antiviral agents for postinfection treatments. Among those several antiviral medications, favipiravir (FAV), remdesivir (RDS), and molnupiravir (MLP) showed a breakthrough in COVID-19 treatments [3].

**Figure 1:**
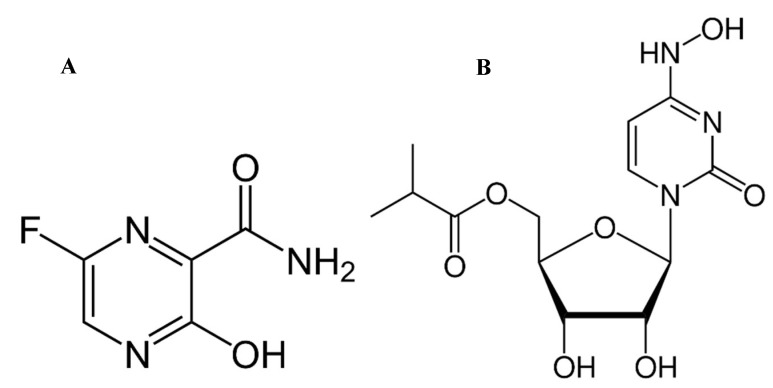
Chemical Structures of (**A**) FAV and (**B**) MLP.

FAV is a prodrug activated by phosphorylation in vivo to produce the active form that inhibits viral RNA polymerase [7]. Several clinical trials were performed on FAV use in COVID-19 infections and declared its efficacy against SARS-CoV-2 in enhancing viral clearance from the body and improving the chest CT of infected patients [8,9,10]. MLP is another broad spectrum antiviral prodrug that is quickly anabolized in vivo into the active triphosphate form, which inhibits viral RNA polymerase that is required for the viral replication cycle [11]. FAV was first developed for treatment of Ebola viral infections [12]. MLP development started in 2013 and focused on targeting alphavirus infections caused by VEEV (Venezuelan equine encephalitis virus). However, both drugs (Chemical Structures Figure 1) were recently modified to target COVID-19 infections. While FAV had already been approved as a COVID-19 antiviral in several countries, MLP had completed phases I and II clinical trials [7,11]. The final results of phase III studies on MLP demonstrated the effect of MLP in significant reduction of death and patient hospitalization risks of nonhospitalized COVID-19 patients [13]. MLP exhibited a wide distribution to mice organs including the lungs and CNS causing a high mutation threshold of the viral genome where it becomes lethal to the virus [14]. Recent human phase I clinical studies indicated MLP’s tolerability and safety, while phases II/III showed its effectiveness against SARS-CoV-2 in mild to light–moderate cases, but not in late–moderate and severe infections [15].

A recent study examined the antiviral effect for the combination of FAV and MLP in SARS-CoV-2 Syrian hamster infections as a model [16]. The combined medication was introduced twice daily to the Syrian hamster infected model. This combination showed a reduction of virus titers in infected lungs by ∼5log10 factor, a complete removal of the virus from more than 60% of infected lungs, and, moreover, the combination therapy prevented the viral transmission to cohoused, nontreated sentinels [16]. Therefore, the study suggested the design of human clinical studies for this combination regimen.

A literature review revealed that a few papers have reported on the determination of FAV including the LC determination of FAV in plasma [12,17,18,19,20], in pharmaceutical dosage forms [21,22], and two spectrofluorometric methods [7,23]. Meanwhile, only two research articles were validated for MLP determination together with its active metabolite in human plasma using LC–MS/MS [24,25]. Up until the writing of this manuscript in December 2021, no conventional method had been reported for the determination of MLP alone or in combination with FAV. Because both drugs are orally bioavailable, there is a high potential for developing an oral single dose multidrug combination pharmaceutical form of FAV and MLP after executing the human clinical studies.

One of the reasons for the development of new analytical methodologies is to establish a reliable, but less expensive and/or less time consuming approach that fits the purpose for which they are required [26]. This research study is aimed at developing new green and simple methods that can estimate simultaneously FAV and MLP in order to be cost-effective in the quality control of the drugs under study in their expected combined dosage forms. These methods consider green analytical chemistry (GAC) principles to minimize the growing ecological impacts of research methods but without loss of the analytical efficiencies using modified approaches for conventional techniques [27]. The methods also consider the economic aspects of pharmaceutical research and quality control laboratories of developing countries to encourage their social responsibilities to the world’s environment during the massive application of drug product analyses.

## 2. Experimental Section

### 2.1. Instrumentation and Software

Chromatographic separation was performed on Agilent Technologies 1200 series chromatographic apparatus equipped G1354A isocratic quaternary pump with online Agilent G1322A vacuum degasser, autosampler injector, and 100 µLvolume injection loop using UV lamp and G1315D photodiode array detector (DAD) connected to Agilent Chemstation software (Agilent Technologies, Santa Clara, CA, USA). RP-C18 was used as stationary phase using Kinetix^®^ column (5 μm, 150 × 4.6 mm), purchased from Phenomenex, CA, USA. pH adjustment was performed on Jenway pH meter model 3510 (Jenway, Staffordshire, UK).

Spectrophotmetric determinations were operated on double beam Schimadzu spectrophotometer (model UV-1201 by Schimadzu, Kyoto, Japan). Samples were estimated in 1 cm quartz cells, and data were manipulated on UV Probe software version 2.43. Matlab 8.2.0.701 (R2013b) software was used to handle chemometric models. For PLS-1 and GA–PLS-1, PLS-toolbox software version 2.1 was utilized.

Dissolution testing was performed using Dissolution apparatus (type PTW II) by Pharma Test, Germany.

### 2.2. Materials

FAV and MLP analytical standards were kindly supplied by the Egyptian International Pharmaceutical industry Co (EIPICo., Nasr City, Egypt), Tenth of Ramadan city, Egypt. Brij-35, SDS, monobasic potassium phosphate anhydrous, and phosphoric acid, all of analytical grades, were purchased from Sigma-Aldrich, Taufkirchen, Germany. Ethanol (EtOH) as HPLC grade and concentrated hydrochloric acid (HCl) were purchased from Fisher Scientific, Newington, NH, USA. Double distilled water was used all over the experimentation and was prepared inhouse.

A placebo solution was prepared by dispersing commonly used tablet and capsules excipients in water at concentrations of 1 mg mL^−1^ using (magnesium stearate, spray dried lactose, carboxymethyl cellulose sodium, titanium dioxide, and maize starch), which were all kindly supplied by EIPICo., Egypt.

Pharmaceutical preparations, Epifluver^®^ tablets (Lot No. 2103597; manufactured by EIPICo., Egypt), and Molcovir^®^ capsules (lot No. MOLCD1003B; manufactured by Optimus, Telangana, India) were kindly supplied by EIPICo., Egypt and were labeled to have 200 mg for FAV and MLP per tablet/capsule, respectively.

### 2.3. Standard Solution Preparation

Separate stock solutions of FAV and MLP were prepared by dissolving 50 mg of each powder in 100 mL 0.1 N HCl to obtain stock solutions of 500 µg mL^−1^. Stock solutions were then used to prepare the working standards. For HPLC method, six linearity standards were diluted in mobile phase at concentrations (0.5, 5.0, 10.0, 20.0, 25.0, and 50.0 µg mL^−1^). Three more quality control (QC) standards were prepared by spiking the drugs under study in the placebo solution at concentrations (5.0, 25.0, and 50.0 µg mL^−1^). Working standard solutions for the chemometric methods were prepared by direct mixing and dilution of the stock solutions using 0.1 N HCl to the required concentrations. Stock and working standards solutions were found stable for 4 days in refrigerator (4–8 °C).

### 2.4. Analytical Procedures

#### 2.4.1. Chemometric Experimentation Using UV Spectrophotometry

A 3-level design (3^2^) was used to construct the calibration and validation models taking into account their linearity ranges and their ratios in pharmaceutical formulation when coadminstered. Twenty-seven FAV and MLP binary mixtures were prepared by mixing different aliquots of the standard stock solutions of FAV and MLP using 0.1 N HCl as diluting medium (Table 1). Scanning of the prepared mixtures was performed to obtain absorption spectra within the range of 210–350 nm with 1 nm interval against a blank of 0.1 N HCl using UV probe software. The spectra were saved as ASCII data files. Absorbencies and concentrations of the mixtures were fed to Matlab software. Optimization of the calibration models was performed. Then, the optimized models were applied to calculate the concentrations of each drug in the chosen mixtures.

#### 2.4.2. Chromatographic Procedure

The chromatographic procedure was established using isocratic micellar mobile phase prepared by dissolving 0.1 M SDS, 0.01 M Brij-35, and 0.02 M monobasic potassium phosphate in 1 L of distilled water, and the pH of the solution was adjusted to 3.1 using dilute phosphoric acid. The mobile phase flow rate was set at 1.0 mL min^−1^. The analytes solutions were injected at 20 µL injection volume and detected by UV detector set at 230 nm. Column temperature was kept at standard room temperature 25 °C.

During experimentation, in between chromatographic runs, the mobile phase was recycled to improve the method’s sustainability. At the end of each working day, the column was purged with a washing mobile phase composed of EtOH:water (50:50, *v*/*v*) for removal of adsorbed surfactants on the stationary phase in order to enhance column’s reproducibility.

#### 2.4.3. In Vitro Dissolution Study

Epifluver^®^ tablets and Molcovir^®^ capsules were tested for dissolution at 37 ± 0.5 °C at the same time as if being coadministered using USP apparatus type-II (Paddle) [28]. Dissolution test was carried out according to USFDA guidelines [29]. Paddles were set at rotation speed 75 rpm. Dissolution medium was composed of 900 mL of 0.1 N HCl (pH 1.2). One Epifluver^®^ tablet and one Molcovir^®^ capsule were simultaneously introduced in the dissolution apparatus. The dissolution medium was carefully covered to prevent evaporation. A total of 5 mL of the dissolution medium was withdrawn at time intervals (3, 5, 7, 10, 15, 20, 30, 45, and 60 min) from the dissolution vessels. The vessels were compensated with fresh medium maintained at 37 ± 0.5 °C, equivalent to the same volume withdrawn each time in order to maintain the total volume. The sampled aliquots were filtered through 0.45 m membrane filters and then diluted using fresh dissolution medium to concentration levels that fell within the range of each method, and then the proposed procedures were followed. The dissolution profiles were constructed by plotting the cumulative percentage of FAV and MLP, which released from their respective dosage form and determined from the regression equations of each method versus time (min) [30].

#### 2.4.4. Pharmaceutical Dosage Forms Analysis Procedure

Five tablets/capsules from each pharmaceutical dosage form (Epifluver^®^ and Molcovir^®^) were accurately weighed, and average weights were calculated. Tablets were then finely powdered before mixing together the combined powders. The capsule contents were mixed thoroughly. An average weight of one tablet and one capsule were transferred into 200 mL volumetric flask, and then the volume was completed to mark using 0.1 N HCl. The content was sonicated for 10 min and then filtered. The obtained solution was serially diluted to obtain the proper concentration for each method. Then, the general procedure for each method was followed.

## 3. Results and Discussion

### 3.1. Chemometric Models

**Figure 2:**
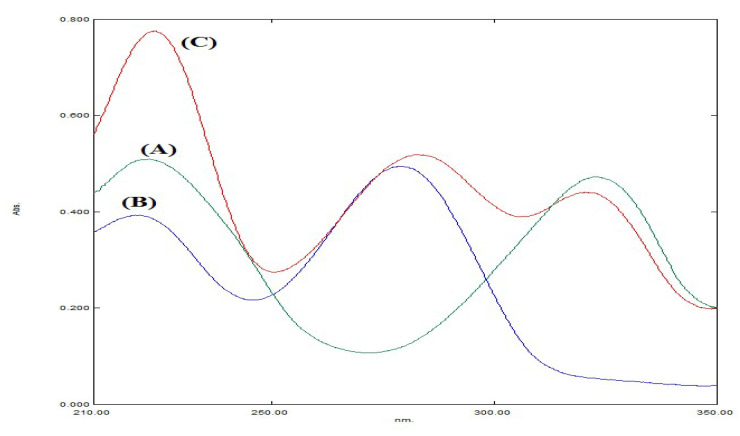
Absorption spectra of (A) 10 µg mL-1 FAV, (B) 10 µg mL-1 MLP, and (C) their mixture.

Scanning the spectra of FAV and MLP revealed great similarity and overlap, especially in the region from 210 to 250 nm. This similarity disabled their simultaneous determination using the direct univariate methods (Figure 2). Models were tried with less spectral points, however, were found to have lower predictive capabilities for one of the two drugs or both. Hence, the ignorance of a part of this spectral zone was avoided. The utilization of several spectral data points, not a single wavelength, was very useful in resolving the complex spectra of the cited drugs. Therefore, four multivariate chemometric models (CLS, PCR, PLS-1, and GA–PLS-1) were applied in this mixture resolution. The prediction capabilities of the four models were evaluated and compared.

#### 3.1.1. Wavelength and Spectral Zone Selection

The overlap of the two selected drug spectra was noticed along the spectra from around 200 nm to points near 350 nm (Figure 2). Different trials using different spectral zones were performed to build the suitable model. Different wavelength ranges such as (200–350), (210–350), (220–350), (230–350), (210–330), (210–350), (210–370), and (210–400) nm were used separately in the construction of the developed models. It was noticed that the spectral points less than 210 were noisy, and the spectra in the region from 200 to 210 nm were serrated. After 210 nm, the spectra were regular. So, the region (200–210) nm was excluded, and the 210 nm spectral point was chosen as start of the selected spectral zone. In respect to the selection of the last spectral point in the selected zone, different wavelengths were tried around 350 nm. It was noticed that the spectral points more than 350 had the lowest absorbance values (near zero absorbances). Hence, the region from 350 to 400 nm did not affect the performance of the constructed models and could be excluded. However, the spectral points less than 350 had important absorbance values that affected the model’s performance especially in respect to FAV prediction as FAV had a characteristic absorbance peak at 325 nm, which could not be excluded. So, the last spectral point should not be less than 350 nm. The selected spectral zone was from 210 to 350 nm at 1 nm interval to obtain 141 spectral points.

#### 3.1.2. Calibration Matrix Construction

The construction of the four developed models was performed using multilevel multifactor design depending on the selection of the spectral zone and the suitable spectral mode [31]. The binary mixtures had different ratios of FAV and MLP to collect as much information as possible about the binary mixture spectra. Data manipulation of spectral points was performed using Matlab software. Eighteen mixtures were chosen to construct the calibration matrix (18 × 141), and nine independent mixtures were utilized as a validation matrix (9 × 141) to experience the predictive capability of the constructed models (Table 1).

**Figure 3:**
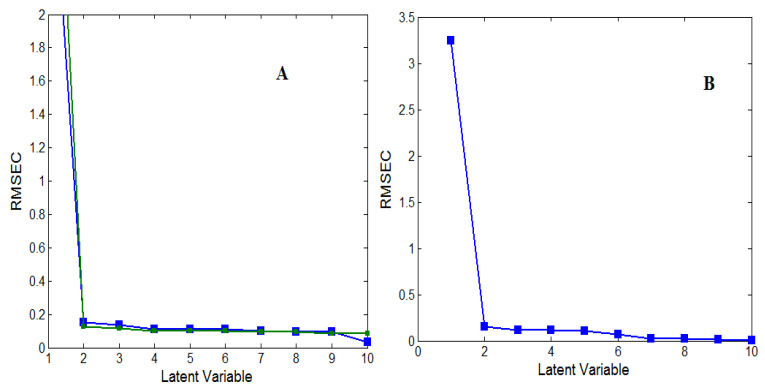
Cross-validation plot of the (**A**) PCR and (**B**) PLS-1 models for FAV and MLP.

For the CLS model, the absorptivity matrix (k-matrix) was constructed using information from the eighteen calibration set mixtures. For PCR and PLS-1 models, cross-validation prestep was applied to select the optimum latent variable number [32]. Two latent variables were the optimum number for both drugs due to having the minimum prediction error values (Figure 3).

For the GA–PLS-1 model, the wavelength selection using a genetic algorithm tool was implemented on the developed PLS-1 model to enhance its predictive capability [33]. GA was performed on 141 variables of the PLS-1 model resulting in a reduction of large spectral point number to 50.35% for FAV and 64.54% for MLP from their original data point number. The optimized GA parameters are presented in the Supplementary Materials (Figure S1) and Table 2.

#### 3.1.3. Model Validation and Evaluation

**Figure 4:**
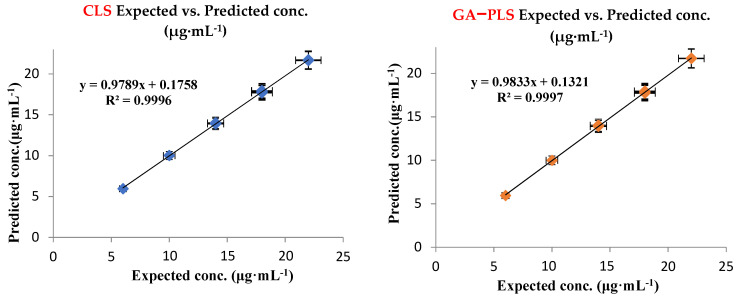
Actual against predicted concentration plots of FAV for CLS and GA–PLS models.

A validation set of nine independent binary mixtures was utilized for model validation by estimating the predicted FAV and MLP concentrations and recoveries in each mixture (Table 3). All predicted concentration values, their percentage recoveries, and standard deviations were found to be satisfactory. Another diagnostic tool was calculating the values of the Root-Mean-Square Error of Calibration (RMSEC) and Prediction (RMSEP). The values of both types of errors did not exceed 0.3 indicating the excellent accuracy and precision of the developed models (Table 3 and Table 4). Graphs of the actual validation set mixture concentrations against the predicted ones were also constructed, and correlation coefficients (r) values were estimated (Figure 4).

Their values were more than 0.9997 proving the excellent agreement between the actual and predicted concentrations and good linearity relationship (Table 4). Graphs of the concentration residuals versus the predicted ones were also constructed (Supplementary Materials Figure S2). Residual graphs had random distribution around zero line indicating the good model construction. All the characteristic parameters of the four developed multivariate models are illustrated in Table 4.

### 3.2. Chromatographic Validation

**Figure 5:**
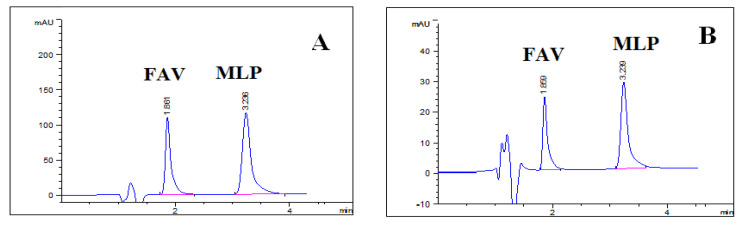
Chromatogram showing separation of drugs under study in (**A**) QC standard (25 µg mL^−1^) and (**B**) combined Epifluvir^®^/Molcovir^®^ dissolution testing under the proposed chromatographic conditions.

The HPLC method was validated according to FDA guidelines [34].The specificity of the proposed method was proven by the good resolution between FAV and MLP peaks as demonstrated in Table 5 and the absence of interferences from excipients (Figure 5). System suitability test parameters of the chromatographic method including retention time (Rt), resolution between analytes (Rs), column efficiency (number of theoretical plates N), peak symmetry, and selectivity (α) were checked to ensure that the system was working correctly during the analysis. System suitability parameters are presented in Table 5.

Linearity was established across the specified range by plotting the response of the linearity standards against their corresponding concentrations. Linearity standards were injected in triplicates, and average responses were calculated. Table 5 shows the linearity data and correlation coefficients. Limits of detection (LOD) and quantification (LOQ) were calculated as a function of standard deviation of intercept (σ) and the slope of calibration curve (S). The results of the LOD were calculated as (3.3σ/S), and the LOQ was calculated as (10σ/S). The LOD and LOQ for FAV and MLP are presented in Table 5.

The accuracy of the proposed method was tested using the three QC standards at low, medium, and high concentration ranges injected in triplicates. The closeness of the calculated percentage recovery results (presented in Table 5) to the true values proved the validity of the method.

The precision was also estimated using QC standards as repeatability (intra-day) and intermediate precision (inter-days) at three different times within the same day and on three different days. Results as a recovery percentage are presented in Table 5 with acceptable standard deviation results.

Robustness is one important parameter when assessing analytical methodologies to ensure that they can withstand small deliberate changes in the experimental conditions. The method was assessed for small changes in pH and concentrations of SDS and Brij-35 compositions of the mobile phase. The pH was changed at three points: 3.0, 3.1, and 3.2. Changes in surfactant concentrations were (0.009, 0.01, and 0.011 M) and (0.095, 0.1, and 0.105 M) for Brij-35 and SDS, respectively. Two quality control standards (5.0 and 50.0 µg mL^−1^) were injected each time, and changes in the recovery percentages were assessed in terms of RSD%. Table 6 results show that such small changes in chromatographic conditions did not have significant effects on the experimental results.

### 3.3. Method Application and In Vitro Dissolution Study

Dissolution testing is an important step in the evaluation of the orally administered solid dosage forms. It measures the rate at which the drug is delivered and released into dissolution media that can be correlated to in vivo studies. In addition, dissolution testing is a way to verify the uniformity of pharmaceutically produced batches. FAV and MLP are not officially listed in any pharmacopoeial monographs; therefore, the recommended dissolution media and conditions were investigated [29]. The dissolution testing can be accepted in terms of quantity dissolved (Q) of the API within a defined time interval according to the release model of the dosage form: immediate, modified, or sustained release. The acceptance criteria for the immediate release of oral solid dosage forms are Q = 80% in 30 min.

**Figure 6:**
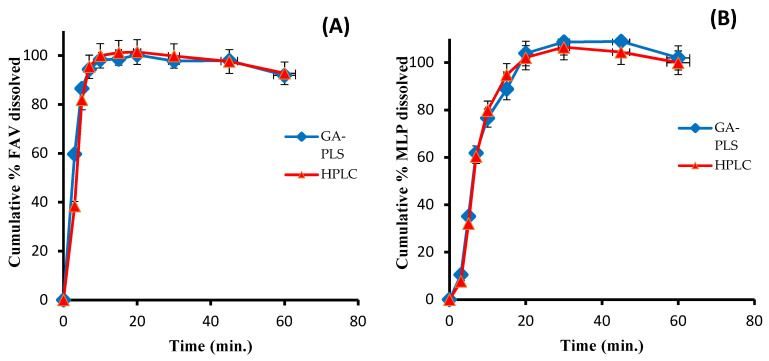
The simultaneous in vitro HPLC and GA–PLS-1 dissolution profiles of (**A**) Molcovir^®^ capsules and (**B**) Epifluver^®^ tablets.

The simultaneous in vitro dissolution profiles of FAV and MLP solid dosage forms were constructed using the proposed analytical methods. The results of both the HPLC and chemometric methods were consistent and revealed that the amount of the simultaneously released drugs increased until reaching a plateau after 15 and 20 min for FAV and MLP, respectively (Figure 6).

### 3.4. Statistical Comparison and Application in the Pharmaceutical Formulation

The developed methods were applied for the assay of Epifluver^®^ tablets and Molcovir^®^ capsules. Good and acceptable results were obtained using the developed methods. Table 7 illustrates the statistical comparison of the obtained results of determination of Epifluver^®^ tablets as representative using the proposed methods versus the results obtained by a reported spectrofluorimetric method [7]. No significant difference was observed among the results (Table 7A). In addition, we show another statistical comparison among the results of the four multivariate developed methods of Molcovir^®^ capsules as a representative example with the developed HPLC method. No significant difference was observed among the results (Table 7B).

### 3.5. Comparative Evaluation of the Developed Analytical Methods

The performances of the developed chemometric methods and their predictive capabilities were compared. Concerning the RMSEP and RMSEC values, GA–PLS-1 was found to have the least error values (Table 4). In addition, GA–PLS-1 surpassed the other three methods in having the highest correlation coefficients (R^2^) and percentage recoveries (Figure 4 and Table 4). From the foregoing parameters, the implementation of GA on the PLS-1 model succeeded in enhancing the results and became the superior choice in comparison with the other three methods.

**Figure 7:**
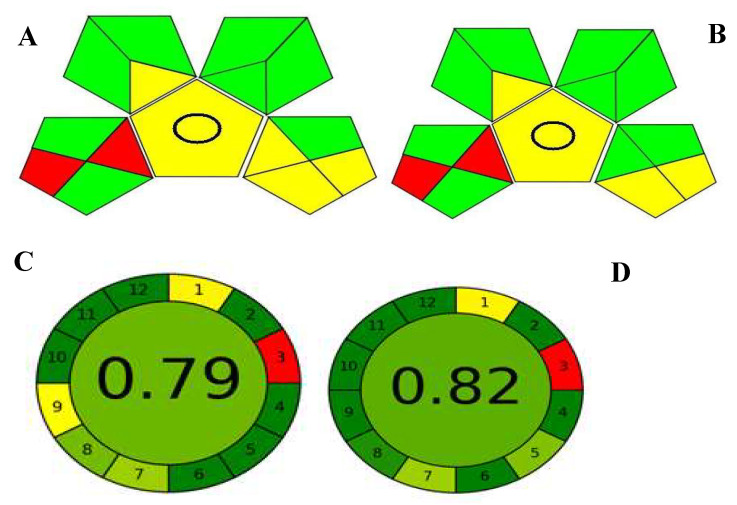
Evaluation of the greenness of the proposed HPLC (**A**) and chemometric (**B**) methods using the GAPI metric, the AGREE metric (**C**) HPLC, and (**D**) chemometric methods.

Being the best developed chemometric model, the GA–PLS-1 was chosen to be compared with the developed HPLC method. HPLC had a lower relative standard deviation between the results of accuracy when compared to the GA–PLS-1. However, the HPLC method, as all routine chromatographic methods, suffers from a higher cost for operation and maintenance, more complex procedure, and longer time for analysis. The two techniques were compared for greenness using the green analytical procedure index (GAPI) [35] and the AGREE metric [36]. The GAPI uses a code with three colors to evaluate the environmental impact: red, yellow, and green. It is composed of 15 pentagrams; each pentagram represents an evaluation of a step of the analytical procedure including sampling, sample preparation, used reagents, instrumentation, and generated waste. Figure 7A,B show the 15 pictograms developed by the GAPI for both developed techniques. As shown, both methods were comparable in nearly all evaluation parameters since both are organic solvent-free methods, have a few simple steps for sample preparation, require no extraction or derivatization step, and have low impact on generated waste. The two methods differ only in the lower right pentagram, which represents the steps of instrumentation energy consumption, waste, waste treatment, and occupational hazards. A spectroscopic analysis showed better impact in terms of the energy required for its instrumentation than the HPLC. The only two red zones in both pictograms represent the offline sampling and transportation required for the QC of any pharmaceutical product since the QC laboratory must be segregated from the production sites according to pharmaceutical regulations. Also, the GA–PLS-1 has better parameters concerning simplicity, cost effectiveness, time saving, and instrument availability.

AGREE uses clock-shaped graphical representation, where the perimeter is divided into 12 zones, each representing one of the GAC principles. The color code is nearly similar to the GAPI ranging from red to yellow and green to indicate ecological impact; however, its colors can range from lighter to darker intensities. AGREE has the advantage over GAPI in providing a numerical estimation of greenness score positioned in the center of its graph [37]. Figure 7C,D represent the AGREE assessment of the studied methods where the spectroscopic method exceeds the HPLC method in the overall calculated score. Both the GAPI and AGREE results were found to be consistent.

Both methods were successfully applied to study the simultaneous dissolution behavior of the cited drugs in their solid dosage forms indicating satisfactory and very close results. The proposed methods succeeded in resolving the interference problems of simultaneous determination of the drugs under study.

In conclusion, the HPLC method will be the best choice when the required issues to focus on are the sensitivity, selectivity, greenness, and availability of well-equipped laboratories. On the other hand, the GA–PLS-1 method will be superior when it comes to the issues of simplicity and instrument availability, especially when used in the routine daily analyses done in low-budget laboratories.

## 4. Conclusions

Two novel methodologies were developed and validated for the simultaneous determination of favipiravir and molnupiravir as a new pharmaceutical combination regimen that was recently reported for activity against SARS-CoV-2 viral infection. The developed methods are sensitive and have high efficiencies; nevertheless, they are totally free of organic solvents. Their greenness was estimated on GAPI and compared to each other in order to prove their low ecological impacts. The methods were successfully applied to in vitro dissolution testing and determination of marketed pharmaceutical dosage forms. The proposed methods are simple and cost-effective; therefore, they are suitable for application in all quality control and/or regulatory laboratories without need for special treatment or expensive instrumentation.

## Figures and Tables

**Table 1:** Concentrations of calibration and validation sets mixtures of FAV and MLP used in the chemometric models.

Sample No.	FAV (μg mL^−1^)	MLP (μg mL^−1^)	Sample No.	FAV (μg mL^−1^)	MLP (μg mL^−1^)
1	14	14	14 *	14	22
2	14	6	15	22	22
3	6	6	16	22	6
4 *	6	22	17	6	18
5	22	10	18 *	18	6
6	10	22	19	6	14
7 *	22	14	20 *	14	18
8	14	10	21	18	18
9	10	10	22*	18	10
10 *	10	18	23	10	6
11 *	18	22	24	6	10
12 *	22	18	25	10	14
13 *	18	14	26	14	18
			27	18	6

* The concentrations of the validation set mixtures.

**Table 2:** Parameters of the developed GA–PLS-1 model.

Parameter	FAV	MLP
Population size	36	36
Maximum generations	34	34
Mutation rate	0.005	0.005
The number of variables in a window (window width)	2	2
Percent of population the same at convergence	100	100
Percent of wavelengths used at initiation	50	50
Crossover type	Double	Double
Maximum number of latent variables	2	2
Cross-validation	Random	Random
Number of subsets to divide data into for cross-validation	4	4

**Table 3:** Validation set results for the four developed chemometric methods.

Mix No.	Actual Conc. (μg mL^−1^)	FAV				Actual Conc. (μg mL^−1^)	MLP			
		CLS	PCR	PLS-1	GA–PLS-1		CLS	PCR	PLS-1	GA–PLS-1
4	6	99.23	99.37	99.37	99.07	22	98.49	98.46	98.46	98.58
7	22	98.32	98.29	98.29	98.57	14	98.94	98.96	98.96	98.44
10	10	100.05	100.09	100.09	99.93	18	97.74	97.72	97.72	97.86
11	18	99.72	99.73	99.72	99.80	22	98.73	98.71	98.72	98.34
12	22	98.70	98.69	98.69	98.85	18	98.87	98.88	98.88	98.36
13	18	98.16	98.14	98.14	98.43	14	99.85	99.85	99.85	99.36
14	14	100.05	100.08	100.08	100.19	22	97.79	97.77	97.77	97.37
18	18	99.15	99.12	99.12	99.44	6	99.17	99.25	99.25	98.67
20	14	99.13	99.15	99.14	99.13	18	98.01	98.00	98.00	97.91
Mean		99.17	99.18	99.18	99.27		98.62	98.62	98.62	98.26
SD		0.69	0.71	0.71	0.61		0.69	0.71	0.71	0.57
RSD		0.70	0.72	0.72	0.62		0.70	0.72	0.72	0.58
RMSEP		0.193	0.195	0.195	0.165		0.280	0.283	0.283	0.217

**Table 4:** Calculated validation parameters for the developed chemometric models.

Parameter	CLS		PCR		PLS-1		GA–PLS-1	
	FAV	MLP	FAV	MLP	FAV	MLP	FAV	MLP
Wavelength	210–350 nm							
Linear range	6.0–22.0 μg mL^−1^							
RMSEC	0.150	0.124	0.150	0.124	0.146	0.120	0.127	0.117
LV number	-	-	2	2	2	2	2	2
Accuracy (%recovery *)	99.45	100.44	99.46	100.45	99.46	100.45	99.67	100.66
RSD (%)	1.58	1.42	1.58	1.42	1.58	1.40	1.40	1.39

* Calculated from the validation set actual and predicted concentration graph for the chemometric methods.

**Table 5:** System suitability and validation results for determination of FAV and MLP under the proposed LC method.

Parameter	FAV	MLP
R_t_ (min) ± RSD	1.87 ± 1.23	3.24 ± 0.78
Resolution	------	7.0
Selectivity (α)	------	3.45
Peak symmetry	0.73	0.81
Theoretical plates (N)	3350	4400
Linear range	0.5–50.0 μg mL^−1^	
Accuracy (%recovery) *	99.99 ± 0.82	99.99 ± 1.23
%Error	0.284	0.490
LOD (μg.ml^−1^)	0.04	0.02
LOQ (μg.ml^−1^)	0.12	0.05
R^2^	0.9999	1.00
Slope	50.9470	30.0777
Intercept	7.3269	−2.8984
**Spiked QC concentration**	Intra-day precision **	
5.0 µg mL^−1^	102.50 ± 1.83	99.46 ± 1.21
25.0 µg mL^−1^	99.54 ± 0.186	98.43 ± 0.07
50.0 µg mL^−1^	100.16 ± 0.15	99.96 ± 0.18
	Inter-day precision **	
5.0 µg mL^−1^	101.67 ± 1.88	99.97 ± 1.73
25.0 µg mL^−1^	99.97 ± 0.63	98.94 ± 0.53
50.0 µg mL^−1^	100.29 ± 0.44	99.85 ± 0.26

* Average recovery% ± RSD% (*n* = 9); ** Average recovery% ± RSD% *(n* = 3).

**Table 6:** Robustness of the proposed HPLC methods for the determination of FAV and MLP.

Parameter	FAV ^a^	MLP ^a^
pH ± 0.1	0.57	1.19
Brij-35 concentration ± 0.001 M	0.69	0.78
SDS concentration ± 0.005 M	1.05	0.89

^a^ Average RSD of percentage recovery results (*n* = 6).

**Table 7:** Statistical comparison and application of the developed methods on the pharmaceutical formulation.

**A: Statistical Comparison among the Results Obtained by Developed Methods and the Reported Method [--] for FAV in Epifluver^®^ Tablets**							
	**Parameter**	**Reported Method [7] ^a^**	**HPLC** **Method**	**CLS**	**PCR**	**PLS-1**	**GA–PLS-1**
	Mean	100.80	100.17	99.31	99.12	99.27	99.12
**FAV**	V	2.25	1.34	1.17	1.16	1.23	0.85
	N	3	5	5	5	5	5
	Student’s *t*-test (t-tabulated 2.447) ^b^	--	0.673	1.650	1.863	1.647	2.003
	F- test (F-tabulated 18.00) ^b^	--	1.68	1.92	1.94	1.84	2.64
**B: Statistical comparison among the results obtained by the developed HPLC method and chemometric methods for MLP in Molcovir^®^ capsules.**							
**Parameter**			**HPLC Method**	**CLS**	**PCR**	**PLS-1**	**GA–PLS-1**
**MLP**	Mean --		100.52	100.94	100.96	101.14	100.98
	V		0.437	0.598	0.639	0.690	0.470
	N		5	5	5	5	5
	Student’s *t*-test (t-tabulated 2.306) ^b^		--	0.923	0.948	1.306	1.080
	F-test (F-tabulated 15.98) ^b^		--	1.37	1.46	1.58	1.08

^a^ Spectrofluorometric method based on determination of FVR in Britton–Robinson buffer of pH 4 at 436 nm as emission wavelength and 323 nm as excitation wavelength. ^b^ Figures in parentheses are the corresponding tabulated values at *p* = 0.05.

## Data Availability

All data are available from the corresponding author upon request.

## Supplementary Materials

The following supporting information can be downloaded at: https://www.mdpi.com/article/10.3390/molecules27072330/s1, Figure S1: Parameters of GA–PLS model for MLP determination; Figure S2: Actual against residual concentrations plots of FAV for CLS and GA–PLS models.
