# Supplementary material for: Two Green Micellar HPLC and Mathematically Assisted UV Spectroscopic Methods for the Simultaneous Determination of Molnupiravir and Favipiravir as a Novel Combined COVID-19 Antiviral Regimen
Source: Molecules. 2022 Apr 4;27(7):2330. doi: 10.3390/molecules27072330 (PMC9000667; doi:10.3390/molecules27072330)
Supplement: Supplementary file 1 [file molecules-27-02330-s001.zip › molecules-1650335-supplementary.pdf]

Supplementary File

Two Green Micellar HPLC and Mathematically Assisted UV Spectroscopic Methods for the Simultaneous Determination of Molnupiravir and Favipiravir as a Novel Combined COVID-19 Antiviral Regimen

Yasmine Ahmed Sharaf [1,†], Sami El Deeb [2,3,*], Adel Ehab Ibrahim [3,4,†], Ahmed Al-Harrasi [3] and Rania Adel Sayed [1]

[1] Analytical Chemistry Department, Faculty of Pharmacy, Zagazig University, Zagazig 44511, Egypt; yasminesharaf2009eg@gmail.com (Y.A.S.); raniaadelsayed@gmail.com (R.A.S.)
[2] Institute of Medicinal and Pharmaceutical Chemistry, Technische Universitaet Braunschweig, Germany
[3] Natural and Medical Sciences Research Center, University of Nizwa, P.O. Box 33, Birkat Al Mauz, Nizwa 616, Oman; adel.ehab@pharm.psu.edu.eg (A.E.I.); aharrasi@unizwa.edu.om (A.A.-H.)
[4] Analytical Chemistry Department, Faculty of Pharmacy, Port-Said University, Egypt
* Correspondence: s.eldeeb@tu-bs.de
† These authors contributed equally to the current work.

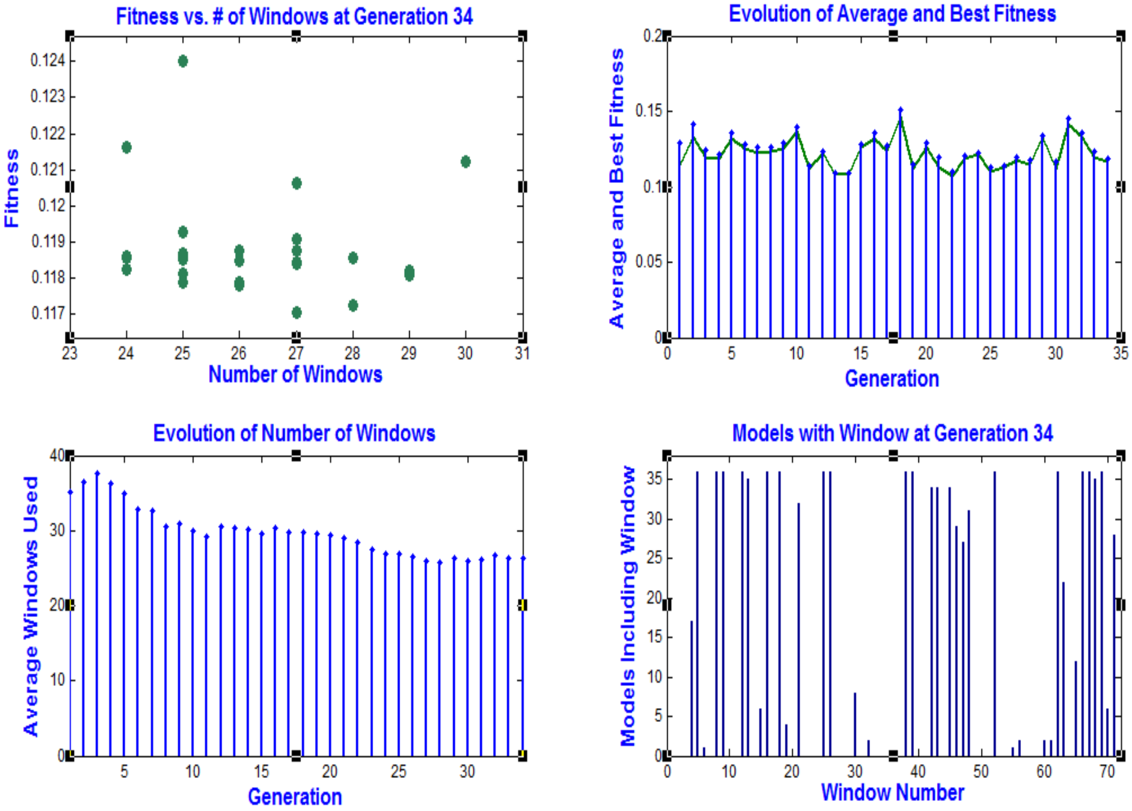

Figure S1. Parameters of GA-PLS model for MLP determination.

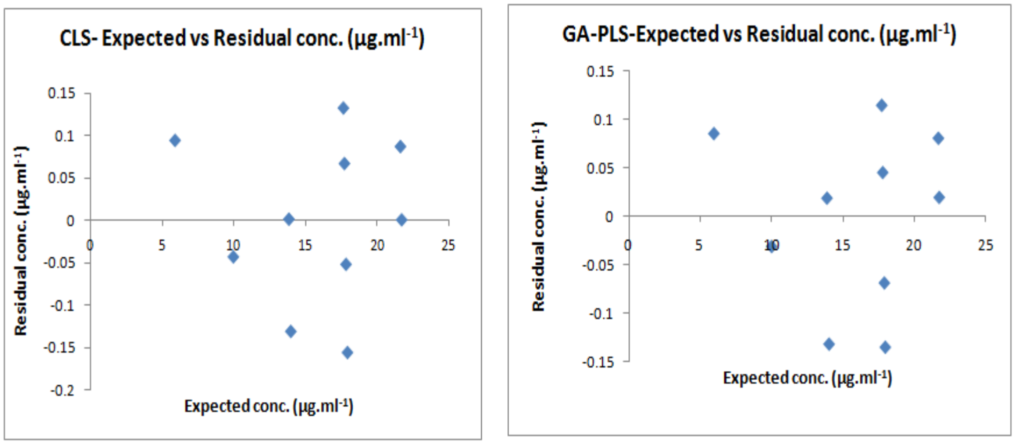

Figure S2. Actual against residual concentrations plots of FAV for CLS and GA-PLS models.
